# Is the health system working for people? Comparison of adolescent and adult perspectives from the people´s voice survey in Germany

**DOI:** 10.1186/s12889-026-30086-5

**Published:** 2026-10-28

**Authors:** Megumi Nagase, Susanne Carai, Mariana Lopes Simões, Ralf Weigel

**Affiliations:** 1 Friede Springer endowed Professorship for Global Child Health, Witten/Herdecke University, Alfred-Herrhausen-Straße 50, Witten, 58455 Germany; 2 World Health Organization Regional Office for Europe, UN City, Copenhagen, Denmark; 3 Graduate School “Health Policy and Systems in Uncertainties” (GRASP), AG3: Epidemiology & International Public Health, Bielefeld University, Bielefeld, Germany

**Keywords:** People´s Voice Survey, Adolescents, Health system, Healthcare experience

## Abstract

**Background:**

People-centred health system performance assessments rely largely on adults, with limited inclusion of adolescents despite their distinct health needs and rights to participate in decisions concerning their health. The People’s Voice Survey is a multi-country, adult-population-representative survey of health system perceptions, but it includes adolescents in the German survey. Germany’s health system offers financial protection and commits to adolescent participation, yet little is known about adolescents’ views of its performance. We aimed to identify adolescent-specific experiences and perspectives by comparing adolescents’ and adults’ experiences and perceptions of health system performance.

**Methods:**

Population-representative data were collected through an online panel between December 2024 and January 2025. Respondents could choose to take the survey in either German or English. Because adolescents were intentionally oversampled to enable age-group comparisons, we conducted a weighted cross-sectional analysis comparing adolescents (15–19 years) with adults (≥ 20 years) using descriptive statistics and chi-square tests. Variables included self-reported health status, confidence and autonomy in managing health, experiences during care delivery, and perceptions of the health system. Weighted descriptive analyses and chi-square tests were performed.

**Results:**

The total sample included 2,698 participants: 257 adolescents and 2,441 adults. Compared with adults, adolescents reported better physical but poorer mental health, lower confidence and autonomy in making their own health decisions and communicating concerns to healthcare providers. Compared with adults, adolescents more frequently perceived medical mistakes and receiving care without feeling sufficiently informed, and reported higher levels of unfair or discriminatory treatment. Adults were more critical of the changes to the health system in the past two years and of the system's overall performance. Despite adolescents’ poorer experiences, they had a more positive outlook on the health system's performance.

**Conclusions:**

Our findings underscore the need to support adolescents' autonomy in making their own health-related decisions and participating in healthcare through age-appropriate communication and opportunities for independent consultations. They also highlight the importance of directly including adolescents in health system performance monitoring, as relying primarily on adult populations may overlook adolescents' unique healthcare experiences and unmet needs.

**Supplementary Information:**

The online version contains supplementary material available at https://doi.org/10.1186/s12889-026-30086-5.

## Background

Growing attention has been paid to adults’ experiences of care, yet adolescents’ perspectives on healthcare quality and responsiveness have received comparatively less attention [1, 2]. However, adolescent representation in health system performance research is critical because adolescence is an important developmental period characterised by increasing autonomy, changing health needs, and long-term effects on health trajectories [3–5]. In addition, adolescents’ negative healthcare experiences may influence their future healthcare-seeking behaviours and trust in health systems [6].

Germany invests substantially in its health system and offers its users strong financial protection. In 2023, the country had the second-highest health expenditure in the European Union (EU), amounting to 12.7% of gross domestic product [7]. Public and compulsory financing accounted for 86% of total health expenditure, while out-of-pocket payments represented just over 11% [7]; the remaining was financed mainly through voluntary or complementary private insurance. Such figures indicate comparatively low financial barriers to healthcare access. However, health outcomes remain modest. Life expectancy in Germany is slightly below the EU average, and avoidable hospital admissions are higher than the EU average for conditions like asthma and chronic obstructive pulmonary disease, diabetes, and congestive heart failure [7]. In parallel, national policy frameworks emphasise investment in child and adolescent health and wellbeing, including the formal involvement of adolescents in the development and implementation of national child and adolescent health strategies [8]. These structural conditions suggest a favourable environment for equitable, responsive, and participatory health care delivery. Nevertheless, understanding adolescents’ experiences remains essential to determine whether these policy goals are being achieved in practice.

The World Health Organization (WHO) defines adolescence as the period between 10 and 19 years of age. This study focuses on older adolescents aged 15–19 years, reflecting the age range covered by the People’s Voice Survey [3].Adolescents in Germany, representing about 9% of the population, reported a substantial number of health complaints, according to the 2022 Health Behaviour in School-aged Children (HBSC) study. For example, more than half of girls and nearly one third of boys among the intentionally selected HBSC age groups (11, 13, and 15 years) reported multiple recurrent health complaints, reflecting significant physical and mental challenges during early and mid-adolescence [9]. These findings underscore the need for a sustained response by the health system and require effective communication, trust-building, safe and adolescent-friendly care models. In addition, adolescents in Germany have a legal right to access health services without parental consent when assessed as mature by the health care provider. Realising this right may enable provider–adolescent engagement and informed decision-making in routine care [10].

Despite well-established policy commitments to youth participation in Germany [11], little is known about how adolescents themselves perceive health system performance. Existing health system experience surveys have predominantly focused on adult populations [12, 13], excluding younger age groups or merging adolescents with adult age categories although international guidance emphasises the incorporation of adolescents’ perspectives and participation in care [14–16]. Whether health systems designed primarily for adult care adequately meet adolescent expectations for respect, safety, communication, and responsiveness therefore remains unclear.

The Quality Evidence for Health System Transformation (QuEST) Network [17] is a global network of academic institutions, governments, and organisations focused on improving health systems worldwide. QuEST developed the People’s Voice Survey (PVS), which offers a unique opportunity to examine health system experiences across age groups using a standardised, population-based approach. To date, it has been implemented in 27 countries globally. The survey also includes non-users of the healthcare system, who are often excluded from patient experience or health responsiveness surveys [18]. By including non-users, the survey can assess barriers to healthcare access and unmet healthcare needs that may not be captured in patient experience surveys. Furthermore, the PVS Germany now allows for comparisons between adolescents and adults within the same health system context across domains such as health status, utilisation of care, care at the last visit and health system confidence. Adolescent inclusion in the PVS could uncover differences between these groups for the first time, support adolescent-responsive health system reforms, and ensure that policy commitments translate into improvements in care delivery, particularly as health and health-related behaviours established during adolescence lay important foundations for health throughout life.

In light of this context, this study aims to identify adolescent-specific experiences and perspectives by comparing adolescents’ and adults’ experiences and perceptions of health system performance in Germany across the following dimensions: health status, healthcare experiences, confidence and autonomy in managing one’s own health, and perceptions of the healthcare system.

## Methods

### Study design

The PVS is a cross-sectional study that collects nationally representative data from both healthcare users and non-users. The survey design, sampling strategy, and data collection procedures have been described in detail elsewhere [19]. The PVS assesses multiple domains of health system performance, including health status, healthcare utilisation, system competence, care experiences during the most recent visit, and confidence in the health system.

### Adaptation of the PVS to the German context

The original PVS was adapted for use in Germany through the following steps:

1. The adaptation of the PVS for the German context was led by MLS and RW and conducted in English. Questionnaire items were reviewed in consultations with researchers at Witten/Herdecke University to assess contextual feasibility and relevance. Changes were kept to a minimum to preserve the core structure and comparability of the original PVS. Items incompatible with the German health care and social system were either adapted or excluded. This included questions on health insurance typology, educational categories, country-specific preventive health examinations, and telephone contact details. Throughout the adaptation process, the Witten/Herdecke University worked closely with a consortium of four European partners and WHO within the context of a European partnership on Transforming Health and Care Systems (THCS) - co-funded by Horizon Europe, concurrently implementing the PVS in four other European countries to ensure cross-national comparability.
2. Translation process: The translation was validated through a structured process. MLS and RW translated the revised (adapted) English survey into German. In parallel, a German-certified translator independently translated the survey from English to German. The two versions were then compared in a consensus meeting and differences discussed until consensus was reached on a final version. A third party from the GESIS Leibniz Institute for Social Sciences [20] facilitated the consensus meeting.
3. Validation of the translation: Cognitive interviews were conducted to validate the translation and assess comprehensibility among adolescents [21]. The cognitive interviews were conducted online by the company IPSOS (Institut Public de Sondage d’Opinion Secteur) [22]. Four adolescents aged 15–18 years (two males and two females) from different regions of Germany were selected via convenience sampling to complete the survey via video call and provide comments on the questions. The feedback was used to refine the survey.

### Study population

Eligible participants were those who: (1) were registered in the online panel we were using (Talk Online Panel) [23] and could access the survey link; (2) resided in Germany; and (3) were able to complete the survey in either English or German. The study included individuals aged 15 years and older to capture the adolescent population aged from 15 to 19 years. Although the World Health Organization defines adolescence as the period between 10 and 19 years of age (young adolescents aged 10–14 years and older adolescents 15–19 years) [24], we included only adolescents aged 15 years and above.

### Sample size consideration

We aimed to recruit up to 2,500 members of the general population, including both adults and adolescents. A sample of 2,000 adult respondents yields a maximum sampling error of ± 1.1%, assuming a 95% confidence level and a population proportion of 50%. A sample of 1,000 respondents provides more than 80% statistical power (α = 0.05, two-sided) to detect a 9-percentage-point difference between two equal groups (e.g., male and female respondents), assuming a baseline proportion of 50%.

### Data collection

The firm Talk Online Panel [23] collected the data between December 2024 and January 2025 by distributing a survey link to registered panellists. Before starting the survey, participants provided informed consent. Talk Online Panel recruited participants using quota sampling, allocating respondents by gender, age, state, household income, and urban/rural setting, except for the oversampled adolescent respondents, to produce a sample representative of the German population. A pilot test involving the first 100 participants (including one adolescent) was initially conducted within the online panel to ensure that the survey tool was functioning as intended. These responses were not included in the final analysis. Talk Online Panel conducted data quality checks and data cleaning according to predefined quality-control criteria, including response behaviour and survey completion time.

### Oversampling of adolescents

For the adolescent group, we conducted additional data collection through the online panel [23] to reach the required sample size. Individuals aged 15–18 years were oversampled by recruiting an additional 208 participants to ensure sufficient statistical power for comparisons between adolescents and adults.

### Analysis

Firstly, we divided the total respondents (*N* = 2,698) into two groups: adolescents aged 15–19, including the oversampled respondents (*n* = 257), and adults aged 20 and over (*n* = 2,441). The data analyst team, which is part of the QuEST Network, cleaned and recoded the data and created initial weights to align the dataset with the PVS data across countries. These weights were modified to be suitable for comparing adolescents and adults. Post-survey weights were calculated using the raking method to align the sample with German Census 2022 distributions for age, sex, education, and federal state [21] to produce representative sample results.

The results were then compared using chi-square tests to analyse the differences between the adolescents’ and adults’ groups. We defined statistical significance at α = 0.05 (two-sided) and conducted all analyses in Stata version 19 [25]. Data are presented as unweighted counts (*n*) and weighted proportions (%).

### Variables

We selected variables representing key dimensions of health system performance relevant to adolescent and adult experiences from the complete PVS Germany. These included self-reported health status, confidence in managing health, experiences during care delivery, and perceptions of the healthcare system.

#### Self-reported health status

Health status was self-reported using separate questions on general health and mental health, each measured on a five-point Likert scale ranging from excellent to poor. In addition, respondents were asked whether they had any longstanding illness or health problem using a binary (yes/no) response. Longstanding illness was defined as a physical or mental health problem that had lasted or was expected to last for six months or longer.

#### Confidence and autonomy in managing one’s own health

All respondents were asked how confident they are in their ability to make decisions about their own health, as well as how confident they are in communicating concerns to healthcare professionals, even when not directly asked. Responses were measured on a four-point scale ranging from very confident to not at all confident.

#### Experiences during care

Respondents who had at least one healthcare visit during the past 12 months were asked whether they had experienced any of the following during their care: perceiving that a medical mistake had been made (with examples such as wrong diagnosis, incorrect medication dosage, or inappropriate sharing of private health information), feeling unfairly treated or discriminated against by healthcare workers, perceiving that physical privacy was not adequately protected during care (e.g. unauthorised persons could overhear a confidential conversation), and receiving treatment without feeling sufficiently informed about it. All responses were recorded as binary yes/no variables.

#### Perception of the health system

To assess trust in and perceptions of the German health system, respondents were asked two questions. The first question assessed perceptions of changes in the health system over the previous two years, with response categories of getting better, staying the same, or getting worse. The second question assessed perceptions regarding the need for change in the healthcare system. Response options included that the system works pretty well and requires only minor changes, that it has some good aspects but requires major changes, or that the system has so many issues that it needs to be completely rebuilt.

### Ethics

The Ethics Committee of Witten/Herdecke University approved the study in December 2024 (No. 330/2023).

## Results

### Sample description

The overall response rate was 19.6% for the total responses, and the median survey completion time was 13 min (range: 4 min–3.6 h for adolescents and 4 min–8.6 h for adults). A total of 2,631 (97.5%) respondents completed the survey in German, and 67 (2.5%) in English. Table 1 summarizes the characteristics of the unweighted sample, along with the weighted proportions and 95% confidence intervals. The supplementary file illustrates the demographic variables that are not included in Table 1.

**Table 1:** People’s voice survey Germany: sample characteristics

Demographics	Adolescents n (weighted %) [95% CI]	Adults n (weighted %) [95% CI]
	*n*=257	*n*=2,441
Gender		
Male	66 (30.1) [21.6-40.1]	1,171 (50.3) [45.9-49.9]
Female	190 (70.0) [68-79.1]	1,265 (49.7) [47.2-52.2]
Other	1 (Not estimated)	5 (Not estimated)
Age in years (weighted mean)	16.9 (16.7-17.1)	50.9 (50.0-51.8)
Born in Germany		
Yes	232 (90.2) [82.3-94.8]	2,251 (92.0) [90.4-93.3]
No	25 (9.8) [5.2-17.7]	190 (8.0) [6.7-9.6]
Have health insurance		
Yes	254 (98.2) [91.6-99.6]	2425 (98.9) [97.0-99.6]
No	3 (1.8) [0.4-8.4]	16 (1.1) [0.4-3.0]
Enrolled health insurance type		
Statutory health insurance	160 (73.9) [63.2-82.4]	2,132 (89.4) [88.0-90.6]
Private health insurance	43 (20.0) [12.7-30.1]	249 (9.3) [8.1-10.6]
Other entitlement (e.g. foreign health insurance)	2 (0.3) [0.1-1.2]	16 (0.6) [0.3-1.0]
Other	10 (5.8) [2.2-14.6]	17 (0.8) [0.4-1.3]

Weighted estimates were not estimable for the “Other” gender category because sampling weights were unavailable for these participants

Among adolescents (*n* = 257), 190 (70.0%) were female, and 66 (30.1%) were male. Nearly half of the adolescents were 18 years old. The highest completed education level was middle school (Mittlerer Schulabschluss) (91, 14.5%). Most of the adolescents (241) were unaware of their family’s monthly household income. Among the insured (98.2%), 73.9% were enrolled in statutory health insurance, the public health insurance system in Germany, which is primarily financed through contributions shared between employees and employers. Most adolescents are covered as dependents under their parents’ health insurance.

The adult group (*n* = 2,441) consisted of almost equal numbers of men and women. Most adults were born in Germany (2,251, 92.0%) and lived in urban areas (1,051, 42.0%). The largest proportion of the monthly household income group fell into the €2,000-€4,000 per month category (980, 39.0%). Regarding education, the highest proportion had higher education degrees (748, 20.6%), followed by those who had completed an apprenticeship or vocational training in Germany’s dual system (589, 16.9%). Among insured adults (98.9%), 89.4% (*n* = 2,132) were covered by statutory health insurance. The proportion of insured respondents was similar in both age groups (98%).

### Self-reported health status

**Fig. 1:**
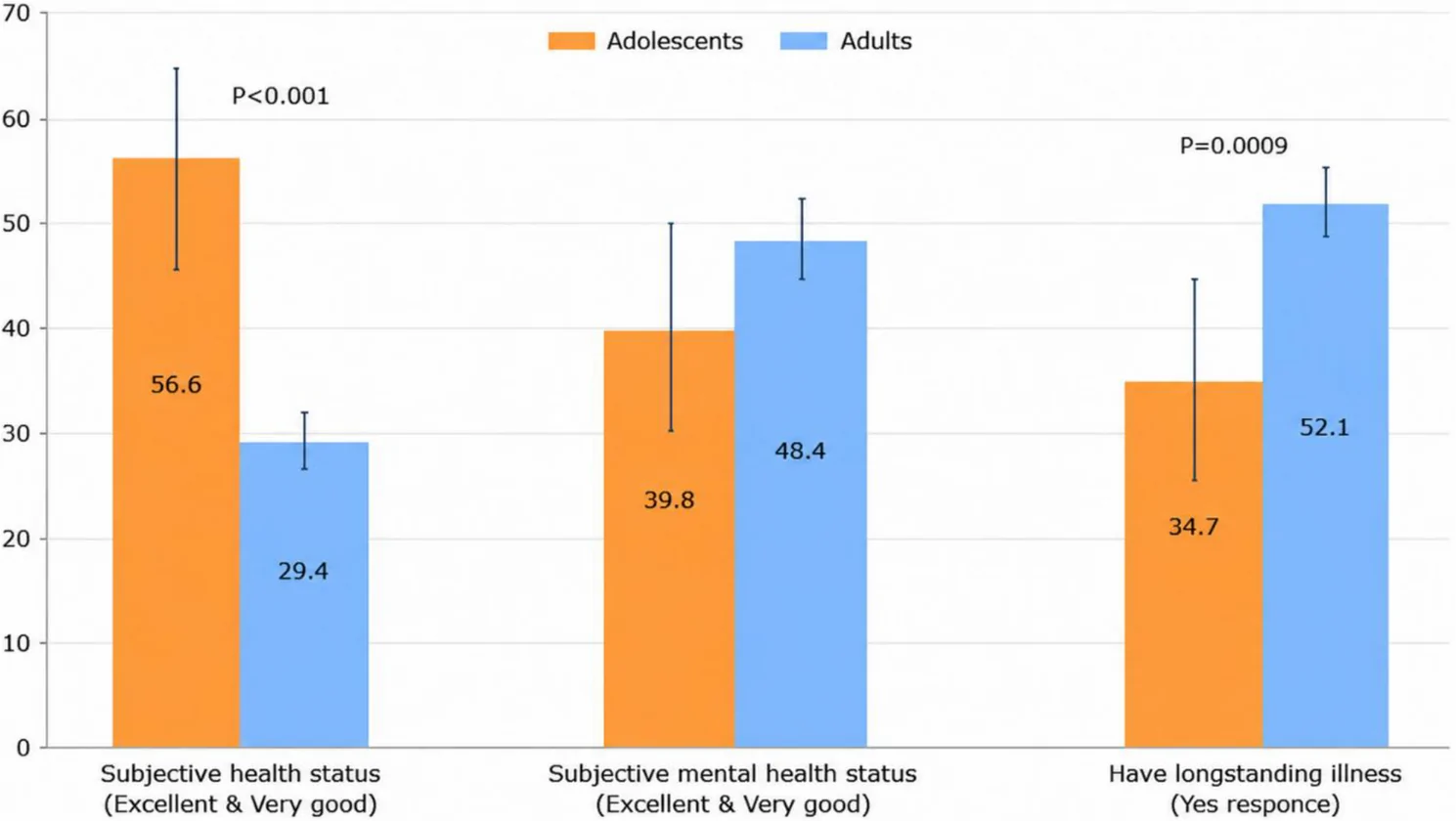
Self-rated health status and mental health among adolescents and adults (%). Weighted proportions of adolescents and adults reporting self-rated health status (95% CI): comparison between adolescent (*n*=257) and adult (*n*=2,441) groups and respective 95% confidence interval

Adolescents rated their physical health significantly better than adults (Fig. 1), with 141 (56.6%) reporting excellent or very good physical health compared with 923 (29.4%) adults (p = < 0.001). In contrast, adolescents reported poorer mental health, with only 39.8% rating their mental health as excellent or very good compared with 48.4% of adults, and a higher rating of poor mental health (23, 10.8% vs. 74, 3.2%) (*p* = 0.097). Adolescents were also less likely to report a longstanding illness or chronic health problem (86, 34.7% vs. 1,211, 52.1%) (*p* = 0.0009). No significant gender differences were observed in either group across these measures, except for mental health status among adults. Adult women were more likely to report poor or fair mental health, whereas positive ratings (very good and excellent) were more commonly reported among men (*p* = 0.0075).

Poor/fair mental health was significantly associated with poor/fair general health in both adolescents and adults (both p = < 0.001). However, the association was stronger among adults. Among respondents reporting poor/fair mental health, 73.3% of adults also reported poor/fair general health, compared with 38.9% of adolescents.

### Confidence and autonomy in managing one’s own health

**Fig. 2:**
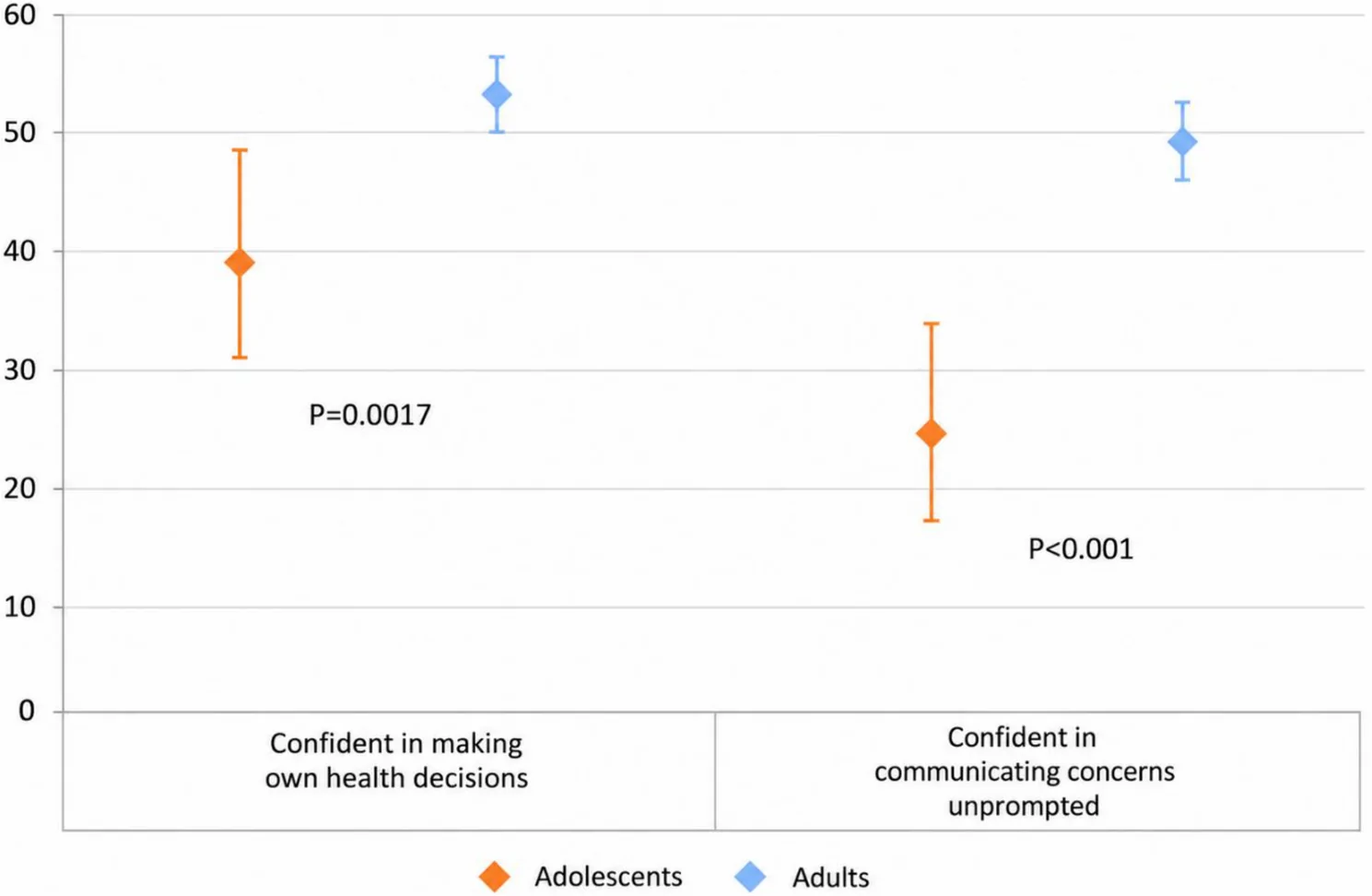
Confidence and autonomy in health decision-making and communicating health concerns when talking to healthcare providers (% of Yes response). Weighted proportions of adolescents and adults reporting confidence and autonomy in health decision-making and communication (95% CI): comparison between adolescent (*n*=257) and adults (*n*=2,441)

Adolescents reported lower levels of perceived responsibility/confidence for managing their health compared with adults (*p* = 0.0017) (Fig. 2). While most adolescents felt at least somewhat confident, only 100 (38.8%) reported being very confident, compared with 1,305 (52.9%) of adults. Adolescents were more likely than adults to report being not too confident (33, 15.4% vs. 132, 6.7%), whereas the proportion reporting somewhat confident was similar (121, 45.5% vs. 995, 40.0%).

A similar pattern was observed for confidence in communicating concerns to healthcare providers, even when not asked (*p* < 0.001). Only one quarter of adolescents reported being very confident in raising concerns without being prompted (65, 25.1%), compared with nearly half of adults (1,207, 49.3%). Adolescents reported being less confident more frequently (17.7% vs. 8.6%) and were more likely to select somewhat confident (55.0% vs. 40.6%). No significant gender differences were observed among adolescents. Among adults, women reported less confidence in communicating concerns to healthcare providers than men (*p* = 0.006).

### Experiences during service delivery

**Fig. 3:**
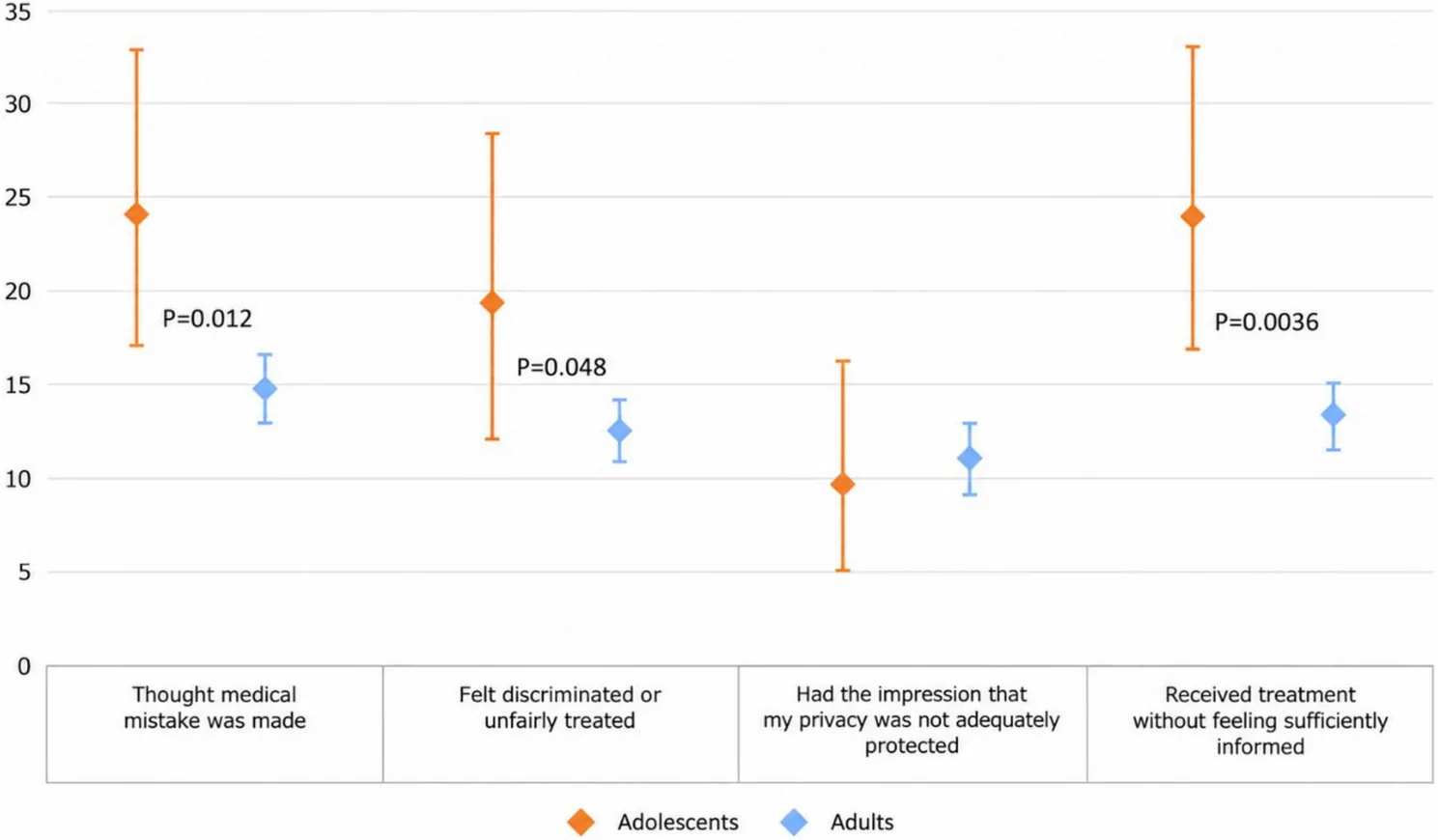
Experiences during service delivery (% of Yes response). Weighted proportions of adolescents and adults who had at least one visit to healthcare facilities in the past 12 months and reported their experiences of service delivery (95% CI): comparison between the groups of adolescents and adults, and their respective 95% confidence intervals

In the 12 months preceding the survey, 96.2% of adolescents aged 15–19 years and 96.0% of adults aged 20 years or older reported at least one healthcare visit. Among those, a higher proportion of adolescents reported perceived medical mistakes in the last 12 months than adults. (62, 24.2% vs. 315, 14.5%, *p* = 0.012), as well as more frequent experiences of receiving treatment without feeling sufficiently informed (63, 23.8% vs. 287, 13.0%, *p* = 0.0036) (Fig. 3). Reports of unfair or discriminatory treatment were also more frequent among adolescents (47, 19.3% vs. 259, 12.2%; *p* = 0.048). Perceived breaches of privacy were similar across the two age groups (28, 9.3% vs. 239, 10.7%, *p* = 0.6719).

Significant gender differences in adolescents’ responses for perceived medical mistakes (*p* = 0.240), unfair or discriminatory treatment (*p* = 0.111), or receiving treatment without sufficient information (*p* = 0.424) were absent. However, adolescent girls were more likely than adolescent boys to report inadequate privacy protection during healthcare encounters (*p* = 0.040). Among adults, women were significantly more likely than men to report perceived medical mistakes (*p* = 0.029), unfair or discriminatory treatment (*p* < 0.001), inadequate privacy protection (*p* = 0.007), and receiving treatment without feeling sufficiently informed (*p* = 0.004).

### Perceptions of the German health system

**Fig. 4:**
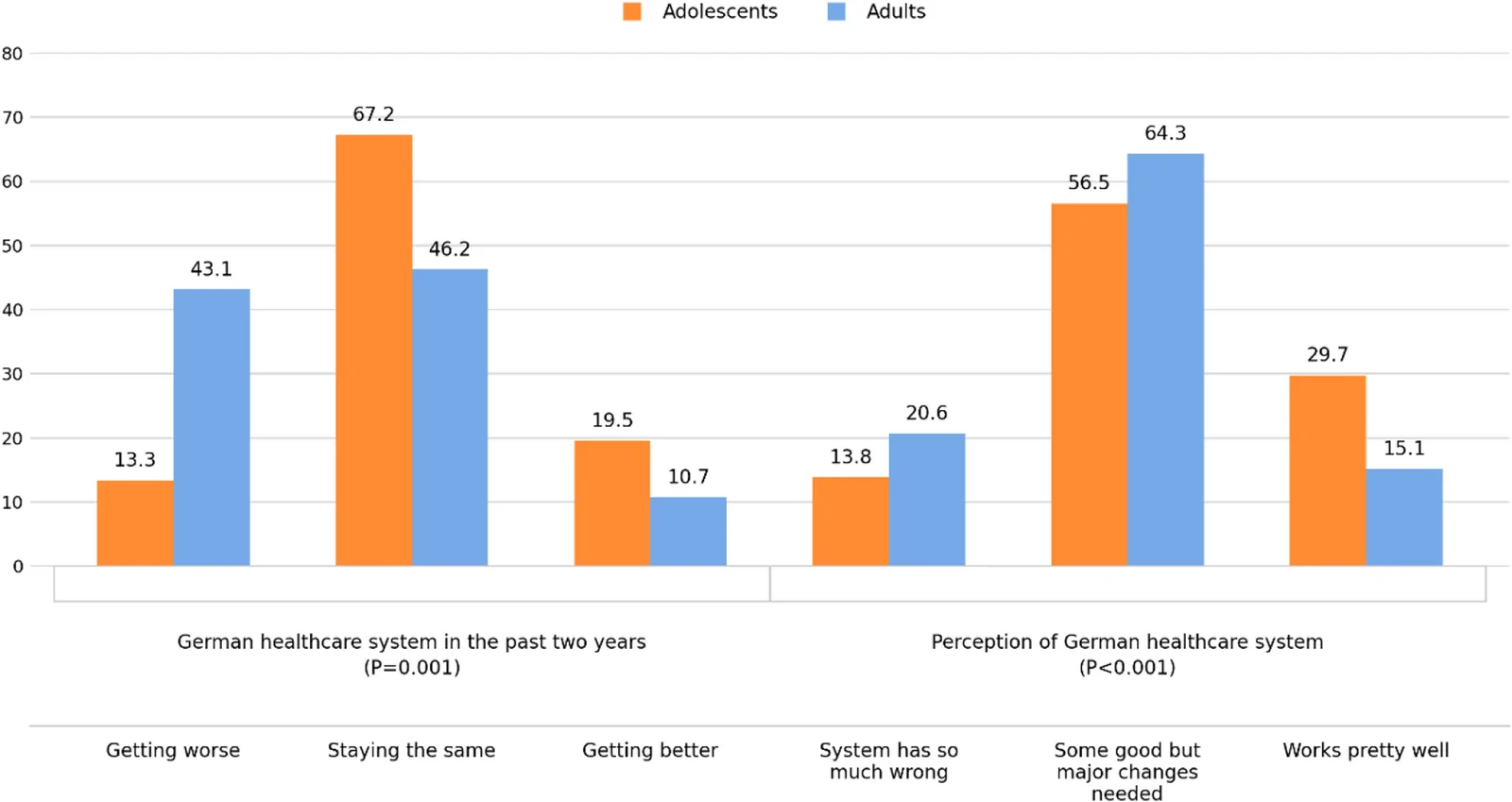
Perception of the German healthcare system among adolescents and adults. Weighted proportions of adolescents and adults reporting perceptions of the German healthcare system: comparison between adolescents and adults

Adolescents generally had more positive perceptions of the national health system than adults (Fig. 4). Regarding the trajectory of health system performance over the past two years, 174 (67.2%) of adolescents perceived the system as “staying the same”, compared with 1,151 (46.2%) of adults. Adults were substantially more likely to report that the system was “getting worse” (1,072, 43.1% vs. 40, 13.3%), whereas adolescents were more likely to perceive the system as “getting better” (43, 19.5% vs. 218, 10.7%, *p* < 0.001). Similarly, adolescents expressed more favourable overall assessments of the health system. Nearly one-third of adolescents (63, 29.7%) reported that the system “works pretty well” compared with 345 (15.1%) of adults. In contrast, adults were more likely to indicate that while the system had “some good” aspects, it needed “major changes” (1,625, 64.3% vs. 165, 56.5%). Adults were also more likely to state that the system “has so much wrong with it” that it needs to be completely rebuilt (471, 20.6% vs. 29, 13.8%, *p* = 0.001).

Significant (*p* < 0.001) gender differences were observed in adults for two health system indicators: perceived health system performance and the trajectory of the health system performance over the two years. Adult women tended to report more favourable perceptions of the health system, whereas men were more critical. No significant gender differences were observed among adolescents.

## Discussion

We compared adolescent and adult experiences of health system performance in Germany across the key dimensions of self-reported health status, confidence and autonomy in managing their own health, experiences during care delivery, and perceptions of the healthcare system. Our findings suggest that adolescents have lower confidence in making health decisions and raising health concerns with healthcare professionals when not prompted. They also report more negative experiences during care delivery while holding a more optimistic view of the health system than adults. In contrast, adults tend to be more critical. This mismatch between adolescents’ personal healthcare experiences and their trust in the system appears contradictory. This apparent contradiction may reflect differences in how adolescents and adults perceive and evaluate specific healthcare experiences and the health system more broadly. These findings underscore the importance of including adolescents’ perspectives in health system quality and performance monitoring.

Adolescents generally rated their physical health more positively than adults, whereas positive ratings for mental health were lower. This divergence is noteworthy because, among adults across 18 countries, poor mental health was strongly associated with poor self-rated physical health [26]. If a similar relationship applied to adolescents, their less favourable mental-health ratings would be expected to coincide with substantially less favourable physical-health ratings. The comparatively positive physical-health ratings observed among German adolescents may therefore indicate that mental and physical health are less closely aligned in this age group. At the same time, adolescents’ poorer mental-health ratings are concerning in their own right. These ratings are consistent with WHO HBSC evidence of deteriorating mental well-being among successive adolescent cohorts, indicating that today’s adolescents experience poorer mental health than both adults and previous generations of young people [27]. 

Adolescents generally reported better self-rated health than adults, consistent with evidence that adolescence is typically the healthiest period of life for physical health [28, 29]. However, adolescence is also a developmental period during which many common mental problems first emerge [27], and substantial biological, psychological and social changes may increase vulnerability to psychological distress.

The proportion of adolescents reporting longstanding or chronic health problems in our adolescent sample (35%) appears higher than estimates from wave 2 of the 2014–2017 German Health Interview and Examination Survey for Children and Adolescents (KiGGS) [30]. KiGGS data show a substantially lower prevalence of long-term health constraints (4-5%) and specific health conditions among children and adolescents [31]. Different diagnostic criteria, perceptions of ill health, and differences in age-related health status may explain the discrepancies between PVS Germany and KiGGS.

Our results also suggest that nearly 90% of both adolescents and adults perceived their physical privacy being adequately protected during healthcare encounters. However, adolescents reported lower confidence in raising concerns with healthcare providers than adults. This suggests that protection of physical privacy alone may not be sufficient to ensure that adolescents feel supported in communicating personal or sensitive health concerns [32, 33]. Their involvement may be further reduced when parents are present, as consultations can become parent-led, limiting adolescents’ participation [34, 35]. Developmental factors and age-related power differences between adolescents and healthcare workers may also reduce adolescents’ sense of agency during visits [36]. Limited training in communicating with adolescents, insufficient recognition of their evolving autonomy, and dismissive or judgemental attitudes may discourage adolescents from raising concerns. In a multi-stakeholder survey across Europe and Central Asia, adolescents identified adults’ failure to understand and respond to their needs—particularly in relation to mental health—as an important priority for action [26]. The findings of the nationally representative PVS survey in Germany suggest that these needs are not yet being adequately met by the current health system. Together, these dynamics may explain adolescents’ perceived low levels of confidence, participation and active involvement in managing their health.

Adolescents appear to perceive more medical mistakes and unfair treatment than adults, although our data do not specify the types of mistakes or how respondents defined them. One possible explanation is that adolescents may interpret healthcare differently from adults. Previous studies indicate that health literacy differs according to sociodemographic characteristics, including age, education and socioeconomic status [37–39]. For example, research in Germany found higher health literacy among younger individuals, those with higher education, and those with higher socioeconomic status [38, 39]. Adolescents may also access health information more frequently online, which could influence their expectations of care and increase their awareness of communication problems or unfair treatment [40–42]. However, more frequent contact with the healthcare system, as we expect in adults, may increase opportunities to recognise and report potential medical problems [43]. Alternatively, adults, who typically have more extensive experience with healthcare services, may be more likely to regard certain practices as routine rather than mistakes or unfairness and might therefore be less critical.

Our findings also showed that a higher proportion of adolescents reported receiving insufficient information during their healthcare visit, suggesting potential communication barriers between healthcare professionals and adolescents. One possible explanation is that healthcare providers may not consistently engage adolescents as active participants during healthcare consultations. Although clinical guidelines recommend direct communication with adolescents and opportunities for confidential and developmentally appropriate consultations [26]. These recommendations may not always be routinely implemented.

Consequently, opportunities to directly engage young patients, provide information in youth-friendly language, and support their involvement in health decisions may be missed. Older adolescents may also experience difficulties engaging with healthcare services if paediatric settings are perceived as child-oriented and insufficiently supportive of adolescent autonomy [26]. Effective communication is essential for high-quality care and has been associated with better clinical outcomes, including improved health status, treatment adherence, and patient safety. Hence, more supportive and inclusive patient–provider interactions may help improve adolescents’ trust and motivation to access health services.

The results also highlighted adolescents’ positive views about the health care system’s performance over the past two years, while adults were more critical in their evaluations. One possible explanation is that adolescents are generally less experienced in navigating the healthcare system independently, as adolescence represents a developmental period of transition toward autonomy [28]. This may limit their opportunities to compare changes in healthcare services over time. In addition, differences in familiarity with the healthcare policies and broader health system issues may also contribute to the differing perceptions observed between the two age groups. Despite these differences, the majority of respondents in both age groups call for major changes to the health system, reflecting a shared perception that substantial improvements are needed. Future research should explore which aspects of the healthcare system are considered most in need of improvement by different population groups.

Gender differences were marked in adults, particularly among women reporting negative healthcare experiences, but not in adolescents. However, the relatively small number of adolescent boys may have limited our ability to detect such differences. Otherwise, the finding is consistent with previous research showing that women often report less favourable healthcare experiences than men across a range of healthcare settings [44, 45]. Furthermore, a previous study suggests that women’s symptoms and health concerns are more likely to be underestimated or attributed to psychological causes than those of men, which may reflect gender bias in clinical practice [46]. Consistently, women reported more frequent negative interactions with healthcare providers, including not being believed, being judged, or experiencing discrimination during healthcare encounters [44]. Women—and, even more clearly, adolescents—are more likely to report substandard personal care while nevertheless rating the system more favourably than men. This contrasting pattern may indicate that personal care experiences and overall assessments of the healthcare system are shaped by different considerations. One possible explanation is that some respondents assess the system from a broader perspective than their own experience, taking into account the care provided to family members, other population groups, or society as a whole, as well as the challenges involved in delivering healthcare. While more research is needed to understand these divergences, it is apparent that favourable system ratings by women and adolescents should not be interpreted as evidence that their healthcare needs and experiences are being adequately addressed.

### Strengths and limitations

The adolescent sample (*n* = 257) was substantially smaller than the adult sample, resulting in wider confidence intervals and limiting the possibility of conducting more detailed subgroup analyses. There was an unequal gender distribution among adolescents. The number of adolescent girls was almost three times that of adolescent boys, presumably because adolescent girls were more willing to participate in the survey. Although weighting was applied to adjust for this imbalance, it may still have influenced the findings, especially for adolescent boys. Adolescents younger than 15 years could not be included. Therefore, the representation of the adolescent group is incomplete. The survey relied primarily on closed-ended questions with binary or Likert-scale response options, limiting our ability to explore the reasons and nuances underlying responses. Recall bias is inherent to retrospective self-reporting of healthcare experiences and cannot be entirely ruled out. Differences in healthcare experience may have influenced adolescents’ and adults’ overall perceptions of the health system. Finally, because of the cross-sectional research design, conclusions about reasons for the findings are limited. The findings should trigger further studies, which may benefit from more in-depth investigation using both qualitative and quantitative methods.

Strengths include the design of a population-based health system survey in Germany that is comparable to those of existing and future PVS from different countries. Importantly, PVS Germany incorporates the perspectives of adolescents aged 15–19 years, a group that is rarely visible.

## Conclusions

Adolescents reported more negative healthcare experiences, lower confidence and autonomy in managing their own health and communicating concerns to healthcare providers compared with adults. Despite these challenges, adolescents expressed more positive views of the health system overall. This study provides, to our knowledge, the first nationally representative evidence from Germany that the health system is not fully meeting adolescents’ needs and performs less well for them than for adults across several important dimensions of care. It therefore confirms at population level concerns previously identified mainly through smaller or more localised studies. The findings underscore the need to strengthen adolescent-responsive care by supporting adolescents’ autonomy in making their own health-related decisions and participating in healthcare using age-appropriate and non-judgemental communication, and providing opportunities for confidential and independent consultations. They also demonstrate the importance of including adolescents directly in health system performance monitoring, as relying primarily on adult populations may conceal age-specific healthcare shortcomings and unmet needs of adolescents. 

## Supplementary Information

Supplementary Material 1.

## Data Availability

The original questionnaire and dataset supporting the conclusions of this article are available upon registration in the Quality Evidence for Health System Transformation (QuEST) repository, https://dataverse.harvard.edu/dataverse/pvs.

## Abbreviations

EU: European Union
HBSC: Health Behaviour in School-aged Children
QuEST Network: Quality Evidence for Health System Transformation Network
PVS: People’s Voice Survey
MLS: Mariana Lopes Simões
RW: Ralf Weigel
THCS: Transforming Health and Care Systems
IPSOS: Institut Public de Sondage d’Opinion Secteur
KiGGS: German Health Interview and Examination Survey for Children and Adolescents
MN: Megumi Nagase
SC: Susanne Carai
