# Supplementary material for: Is the health system working for people? Comparison of adolescent and adult perspectives from the people´s voice survey in Germany
Source: BMC Public Health. 2026 Oct 28;26:3095. doi: 10.1186/s12889-026-30086-5 (PMC13715150; doi:10.1186/s12889-026-30086-5)
Supplement: Supplementary file 1 [file 12889_2026_30086_MOESM1_ESM.docx]

**Supplementary material**

Demographics of adolescent and adult respondents (n = 2,698)
